# Coumestrol inhibits autoantibody production through modulating Th1 response in experimental autoimmune thyroiditis

**DOI:** 10.18632/oncotarget.10353

**Published:** 2016-06-30

**Authors:** Xiaojing Jin, Shujun Wang, Xuemin Zhao, Qian Jin, Chenling Fan, Jing Li, Zhongyan Shan, Weiping Teng

**Affiliations:** ^1^ Department of Endocrinology and Metabolism, Institute of Endocrinology, The First Affiliated Hospital, China Medical University, Liaoning Provincial Key Laboratory of Endocrine Diseases, Shenyang, P. R. China; ^2^ Department of Pharmaceutics, School of Pharmacy, Shenyang Pharmaceutical University, Shenyang, P. R. China; ^3^ Present address: Department of Emergency, The First Affiliated Hospital of Hebei Medical University, Shijiazhuang, P.R. China

**Keywords:** autoimmunity, thyroiditis, coumestrol, phytoestrogen, T helper cell, Immunology and Microbiology Section, Immune response, Immunity

## Abstract

Coumestrol is a common phytoestrogen found in plants and Chinese medicinal herbs. Its influences on experimental autoimmune thyroiditis (EAT) were investigated in this study. Female adult CBA/J mice were fed with drinking water containing 1% Tween80 only (Control group), 0.8 mg/l (L group) and 8 mg/l coumestrol (H group) from 6 to 15 weeks of age, respectively. Their serum coumestrol concentrations were determined by high performance liquid chromatography, which were undetectable, 43.70 ± 21.74 ng/ml and 135.07 ± 70.40 ng/ml, respectively. In addition, the mice (n = 14–16/group) were immunized twice with thyroglobulin (Tg) and Freund's adjuvant to induce EAT during the meantime. Although no overt changes in the extent of intrathyroidal mononuclear cell infiltration were shown in the two coumestrol-treated groups as compared with the controls, serum anti-Tg IgG2a, IgG3 and IgG1 titers, ratio of IgG2a to IgG1 and the percentage of T helper (Th)1 cells in the splenocytes were significantly reduced in the L group. Another consistent change was the significantly decreased expression of splenic IFN-γ mRNA after low dose of coumestrol exposure. Uterine weight was also markedly reduced in the mice of L group. These findings suggest that coumestrol treatment may have some beneficial actions against thyroid-specific autoantibody production in the development of autoimmune thyroiditis through suppression of Th1 response due to its anti-estrogenic activity.

## INTRODUCTION

Estrogens have been known to modulate lymphoid cell growth, differentiation, activation and proliferation after binding to intracellular estrogen receptors (ERs) [1-5]. They usually activate cytokine secretion and antibody (Ab) production. Coumestrol is a common phytoestrogen existent in a variety of leguminous plants (e.g. alfalfas) that are food sources for human being and farm animals. It is also rich in some traditional Chinese herbs (e.g., Radix Puerariae) [6, 7]. Coumetrol is known to have ER binding affinity [8, 9]. As estrogens have multiple actions on immune functions, the effects of xenoestrogens may be superimposed upon the endogenous pituitary-gonadal axis and affect autoimmune responses. Coumestrol has been found to suppress the development of lupus, an estrogen-sensitive systemic autoimmune disease [10].

Autoimmune thyroiditis (AIT) has recently reached a population prevalence of more than 5% with female predominance [4]. It usually shows spontaneous remission during pregnancy and becomes aggravated after parturition [4, 11]. There is still lack of effective therapeutic methods for this prevalent disease. Experimental autoimmune thyroiditis (EAT) induced by thyroglobulin (Tg) immunization is a classical commonly-used animal model in the studies of AIT pathogenesis. It also shows a female preponderance in both incidence and severity of the disease [12]. A study by Guo et al. showed that the incidence of EAT and the extent of mononuclear cell infiltration in the thyroid were significantly lower in ovariectomized (OVX) female SD rats than those in intact controls [13]. These findings suggest that female steroids may stimulate the development of AIT. Using the EAT mouse model, the current study examined the effects of coumestrol exposure on the development of thyroid autoimmunity and the potential mechanisms. Our results may contribute to developing new therapeutic and prophylactic strategies for AIT.

## RESULTS

### Weights and overall health

**Figure 1:**
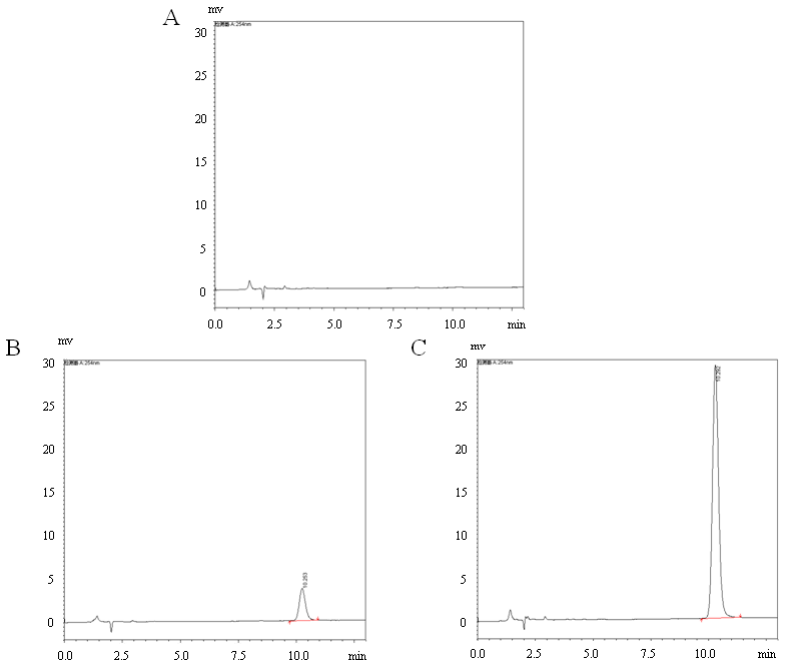
Representative high-performance liquid chromatography (HPLC) profiles in detection of coumestrol dissolved into the drinking water The samples were prepared from the drinking water containing 1% Tween80 only (control group, **A.**), 0.8 mg/l coumestrol in 1% Tween80 (L group, **B.**), 8 mg/l coumestrol in 1% Tween80 (H group, **C.**), respectively. They (*n* = 3/group) were measured by HPLC-UV.

Appropriate dissolution of coumestrol in the drinking water was further confirmed by measurement of coumestrol using HPLC. The concentrations prepared for the H and L groups in the representative samples (*n* = 3/group) were detectable, which were 9.35±0.14 mg/l and 1.17±0.14 mg/l, respectively (Figure 1). The mean amount of water consumed in each of the three groups was 5 ml/mouse/day. No overt difference was found in water consumption among the three groups during this investigation. Successful exposure of the mice to coumestrol was further confirmed by measuring the representative serum samples. Serum concentrations of coumestrol were 43.70 ± 21.74 ng/ml and 135.07 ± 70.40 ng/ml in the representative serum samples of the L and H groups (*n* = 8/group) with significant difference between them (*P* = 0.003), whereas it was undetectable in the sera of the control mice (*n* = 8). The body weight in all groups of female CBA/J mice was monitored until they were sacrificed. There was no markedly change in body weight between coumestrol-exposed mice and the control animals at 6, 8 or 11 weeks of age (data not shown). However, a significant increase in body weight was found in coumestrol-exposed mice at 13 weeks of age ( control group 18.15 ± 0.14g, *n* = 5; L group 21.62 ± 0.84g, *n* = 6, *P* = 0.0001; H group 20.49 ± 0.34g, *n* = 5, *P* = 0.0003) and 15 weeks of age ( control group 19.78 ± 0.46g, *n* = 5; L group 21.80 ± 0.23g, *n* = 6, *P* = 0.019; H group 21.55 ± 0.66g, *n* = 5, *P* = 0.03), implying no obvious toxicity caused by coumestrol exposure at the selected doses. Moreover, there were no overt adverse health or behavior noted in the mice exposed to coumestrol as compared to vehicle controls before the first Tg immunization.

### Coumestrol exposure and the development of EAT

**Figure 2:**
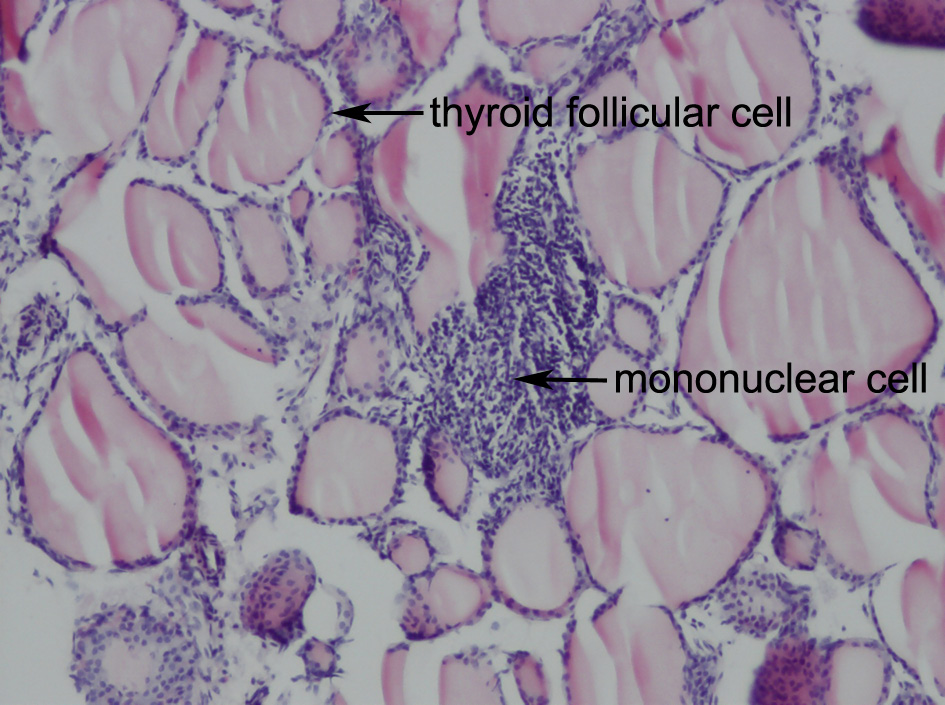
Representative thyroid histology of EAT mice (Original magnification: 100X) CBA/J mice were immunized twice with mouse thyroglobulin (mTg) and Freund's adjuvant at a two-week interval. Thyroid glands were removed, fixed in 10% neutral phosphate-buffered formalin, and stained with hematoxylin and eosin (HE) for histological examination. Prominent mononuclear cell infiltration was accompanied by enlargement and destruction of thyroid follicular cells as the arrow indicates.

To investigate the effect of coumestrol on the development of EAT, we immunized the above coumestrol-treated and control mice with Tg. Following Tg immunization, both intrathyroidal mononuclear cell infiltration and serum TgAb titer were evaluated. There were different extents of mononuclear cell infiltration observed in the mice with EAT, whose scores ranged from 1 to 4 (Figure 2, Table 2). Neither the incidence of EAT nor the mean score for intrathyroidal mononuclear cell infiltration was significantly different between coumestrol-treated mice and control animals.

**Table 2:** The incidence of EAT and the mean score for intrathyroidal mononuclear cell infiltration in coumestrol-exposed mice and the vehicle controls

Group	Incidence	Histological severity of thyroiditis (score/n)						Mean score
		0	1	2	3	4	5	
Control group	86.0%	2	5	3	3	1	0	1.72
L group	75.0%	4	8	1	3	0	0	1.19
H group	66.7%	5	1	7	2	0	0	1.40

Female adult CBA/J mice were randomly divided into three groups. They were exposed to the drinking water containing 1% Tween80 only (Control group), 0.8 mg/l coumestrol in 1% Tween80 (L group), 8 mg/l coumestrol in 1% Tween80 (H group), respectively. All these mice were further immunized twice with mouse thyroglobulin and Freund's adjuvant at a two-week interval. The extent of mononuclear cell infiltration was observed under a light microscope after thyroid sections were stained with hematoxylin and eosin.

**Figure 3:**
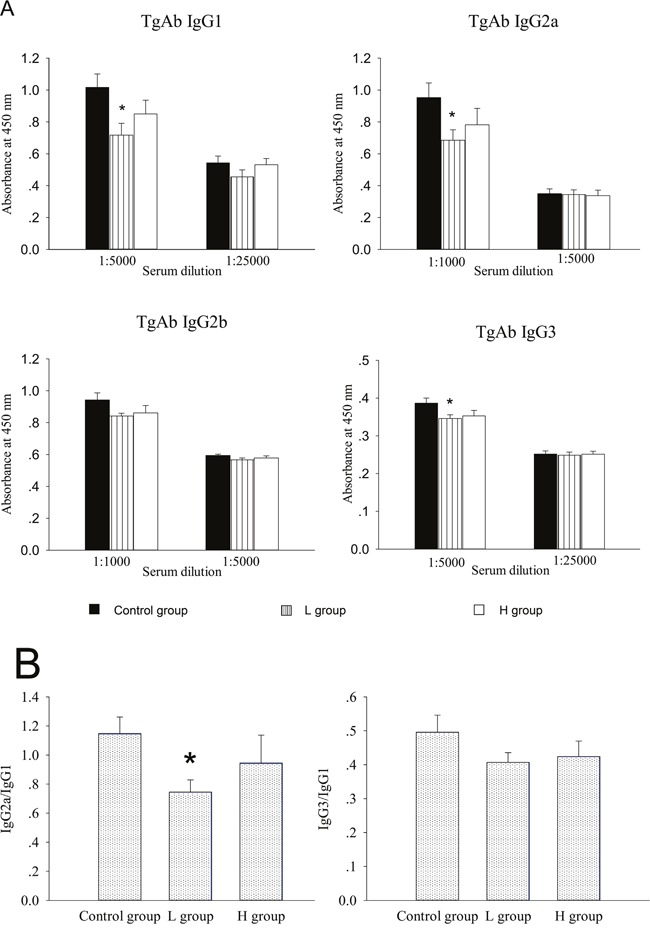
The effect of coumestrol exposure on serum TgAb level in female adult CBA/J mice after induction of EAT These mice had been exposed to the drinking water containing 1% Tween80 only (Control group), 0.8 mg/l coumestrol in 1% Tween80 (L group), 8 mg/l coumestrol in 1% Tween80 (H group), respectively. Serum TgAb level was measured by ELISA. Data are shown as mean ± SD of absorbance at 450 nm (*n* = 10-14/group, **A.**). The ratios of serum IgG2a and IgG3 to IgG1 were further calculated (**B.)** Comparisons between groups were performed using one-way ANOVA followed by Bonferroni's *post hoc* test. *P < 0.05 *vs*. the control group.

Estrogens have been known to enhance antigen-specific Ab response after antigen challenge [1, 3, 5]. Previous studies have reported that coumestrol has ER binding affinity [8, 9, 14]. In this study, the effects of coumestrol exposure on the production of TgAb, a thyroid-specific autoantibody, were assessed. In mTg-immunized mice, serum levels of Tg-specific IgG1, IgG2a, and IgG3 decreased after coumestrol exposure, whereas serum level of IgG2b remained unchanged. There were significant differences between animals exposed to low dose of coumestrol (L group) and the vehicle-treated control animals (Figure 3A). The ratio of serum anti-Tg IgG2a to IgG1 was markedly lower in the L group as compared with that of the control group (Figure 3B). It suggests that coumestrol intake suppresses thyroid autoantibody production in AIT.

### T and B cell responses to coumestrol exposure in the spleens of Tg-immunized mice

**Figure 4:**
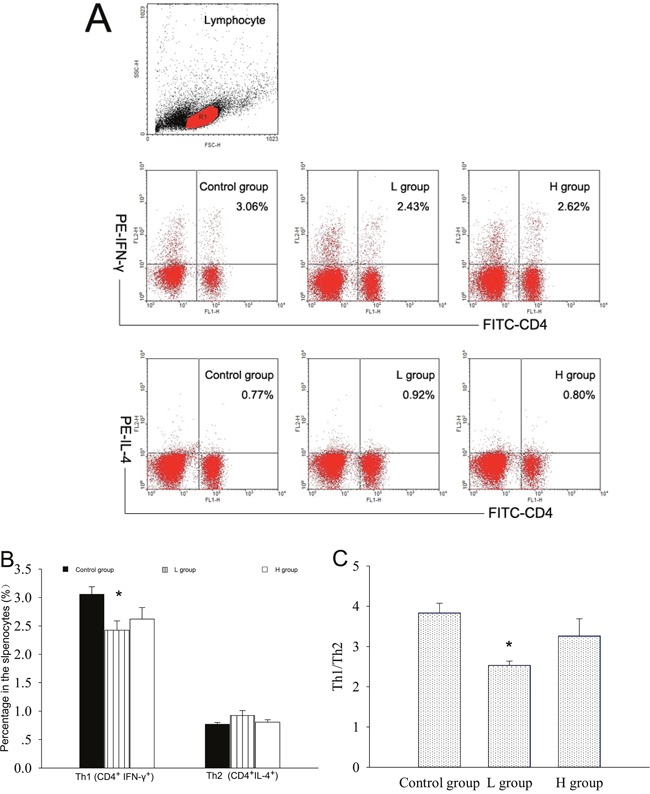
The effects of coumestrol exposure on the differentiation of splenic Th1 and Th2 subsets in female CBA/J mice after induction of EAT These mice had been exposed to the drinking water containing 1% Tween80 only (Control group), 0.8 mg/l coumestrol in 1% Tween80 (L group), 8 mg/l coumestrol in 1% Tween80 (H group), respectively. Their splenic mononuclear cells (*n* = 5 per group) were stained with FITC-labeled anti-CD4 and PE-labeled anti-IFN- γ or IL-4 antibodies, and analyzed on a FACScan flow cytometer as shown in the representative dot plots (**A.**) The percentage of Th1 cells (**B.**) and ratio of Th1 to Th2 (**C.**) were significantly decreased in the L group. *P < 0.05 vs. the control group.

Both T and B cells express ERs, and play important roles in the pathogenesis of AIT [4, 5, 11, 15-16]. The mRNA expressions of T subset-specific transcription factors and typical cytokines in the spleen were investigated. IFN-γ mRNA was less expressed in the spleens of coumestrol-treated, Tg-immunized mice. The difference was statistically significant between the L group and the control animals. IFN-γ can promote the IgG subclass switching to IgG2a and IgG3, which have more opsonizing and complement-fixing abilities than IgG1 in mice [17, 18]. The down-regulation of splenic IFN-γ mRNA was consistent with the changes in serum TgAb as shown above. The mRNA expression of T-bet, GATA3, IL-4, RORγt, IL-17A, Foxp3 or TGF-β was not significantly changed after coumestrol treatment (Table 3). The ratio of IFN-γ/IL-4 mRNA expression level was also significantly lower in the L group than the control animals (control group 2.69 ± 0.34, *n* = 5; L group 1.22 ± 0.41, *n* = 4, *P* = 0.027). No significant difference was shown in the ratio of T-bet/GATA3, RORγt/Foxp3 or IL-17A/TGF-β between the coumestrol-exposed mice and the vehicle-treated controls. These findings suggested that coumestrol exposure may suppress Th1-type response in the development of AIT. It was further demonstrated by the changes in the percentages of splenic Th1 and Th2 subsets (Figure 4). The percentage of Th1 cells (CD4^+^ IFN-γ^+^) in the splenocytes was significantly decreased, while the relative number of Th2 cells (CD4^+^IL-4^+^) was not markedly changed in the L group as compared with that of the control group (Figure 4A and 4B). The ratio of Th1/Th2 was also significantly lower in the L group than the control group (Figure 4C), indicating that coumestrol exposure may inhibit the shift of Th toward Th1 in the spleens of EAT mice.

**Table 3:** The effects of coumestrol exposure on mRNA expressions of Th- and Treg-specific transcription factors and typical cytokines in the spleens of female CBA/J mice after induction of EAT

		Control group	L group	H group
Th1	T-bet	0.82 ± 0.19	0.60 ± 0.19	0.79 ± 0.20
	IFN-γ	4.86 ± 0.77	1.42 ± 1.19 *	3.54 ± 1.62
Th2	GATA3	0.76 ± 0.10	1.02 ± 0.75	0.77 ± 0.24
	IL-4	1.85 ± 0.41	2.47 ± 0.38	2.20 ± 1.33
Th17	ROR-γt	0.17 ± 0.03	0.20 ± 0.08	0.19 ± 0.11
	IL-17	0.12 ± 0.08	0.21 ± 0.08	0.14 ± 0.08
Treg	Foxp3	5.47 ± 1.82	3.91 ± 1.96	4.45 ± 1.22
	TGF-β	0.12 ± 0.04	0.07 ± 0.02	0.11 ± 0.06

Female adult CBA/J mice were exposed to drinking water containing 1% Tween80 only (Control group), 0.8 mg/l coumestrol in 1% Tween80 (L group), 8 mg/l coumestrol in 1% Tween80 (H group), respectively. All the three groups of mice were further immunized with mouse thyroglobulin and Freund's adjuvant for induction of EAT. The mRNA expressions of Th- and Treg-specific transcription factors and typical cytokines in the spleens were examined by quantitative RT-PCR. The transcript levels were assessed using the comparative Ct method, normalized to that of murine GAPDH, and expressed as mean ±SD of △Ct values (n=5-8/group). Comparisons between groups were performed using one-way ANOVA followed by Bonferroni's *post hoc* test. *P <0.05 *vs* the control group.

Plasma cells differentiated from mature B cells (B220^+^sIgM^+^) are responsible for the production of autoantibodies, which are mainly regulated by Th cells. The proportions of B220^+^sIgM^+^ cells in the spleens of coumestrol-exposed, Tg-immunized mice (H group 6.38±1.78%; L group 7.94±2.68%; *n* = 4/group) were not significantly different from that of the vehicle-treated, Tg-immunized animals (5.81±1.18%, *n* = 4).

### Coumestrol exposure and the change of E2 production and weights of ovaries and uteri

Serum concentration of E2, the main endogenous estrogen, was not significantly different between coumestrol-treated groups and the vehicle control. Ovarian weight did not show a significant change after coumestrol exposure, either (Table 4). The potential anti-estrogenic effects of coumestrol on the reproductive organs have previously been reported in adult animals [19]. Uterine weight gain is a typical marker of estrogenic action. It was further investigated in this study. The uterine wet weight was markedly reduced in the L group as compared with that of the control group (Table 4). These results indicated that coumestrol may directly exert its anti-estrogenic activity without altering the production of estrogens.

**Table 4:** The effects of coumestrol exposure on the level of serum E2 and weights of the ovary and uterus

Group	Serum E2 (pg/ml)	Relative ovarian weight (mg/g)	Relative uterine weight (mg/g)
Control group	127.17±23.94	0.17±0.13	3.39±1.09
L group	107.39±51.65	0.11±0.08	2.34±0.76 *
H group	112.70±49.27	0.13±0.09	2.81±0.60

Sera, ovaries and uteri were isolated from those coumestrol-exposed female adult CBA/J mice (L and H groups) and the control animals as described above, respectively. Serum level of estradiol (E2) was determined using an EIA kit (n=10-12/group). The ovaries and uteri (n=8-10/group) were weighed after associated fat and internal fluid was removed. Their weights are presented as relative weights after normalized to body weights using the following equation: organ weight (mg)/body weight (g). Comparisons between groups were performed using one-way ANOVA followed by Bonferroni's *post hoc* test. *P<0.05 *vs.* the control group.

## DISCUSSION

The incidence of AIT in women is three to four times as high as that in men. Okayasu et al. reported that administration of E2 could enhance the production of TgAb in both sham-operated and castrated EAT male mice [20]. Chailurkit et al. investigated the relationship between circulating estradiol and thyroid autoimmunity in men. They compared the difference in circulating E2 based on the test results for TRAb, TPOAb and TgAb. It was found that serum level of E2 was significantly higher in the subjects with positive TRAb [21]. These findings suggest that estrogens can stimulate the development of autoimmune thyroid diseases. Estrogens have pleiotropic effects on the immune system and autoimmune responses *via* two ER subtypes, ERα and ERβ [1, 4, 5, 22]. ERs are widely expressed in the immune cells, including T and B cells [5, 22]. Our previous study has shown that E2 treatment of OVX mice can reduce the percentage of splenic mature B cells, enhance the secretion of IFN-γ from splenocytes, and augment the production of anti-DNP antibody after dinitrophenyl-keyhole limpet hemocyanin (DNP-KLH) immunization [1, 3]. The effects of estrogens, subtype-specific ER agonists, and xenoestrogens on systemic autoimmune responses of lupus have been investigated in the B/W F1 mouse model [2, 23, 24]. E2 could promote the development of lupus disease in terms of shorter survival, accelerated cumulative albuminuria, and more anti-DNA IgG2a and IgG3 production [2]. Phytoestrogens are plant-derived compounds that can interact with ERs [25]. Several kinds of phytoestrogens, including coumestrol, were found to have beneficial effects on lupus [10, 26, 27]. Coumestrol, a benzofurocoumarin, is a naturally occurring plant estrogen with both receptor-dependent anti-estrogenic and estrogenic activities [8, 9, 25]. To our knowledge, there is no report about its effects on the development of AIT. The pathogenesis of this organ-specific autoimmune disease is different from that of systemic lupus.

In this study, we ensured the levels of coumestrol exposure by determining its concentrations in the drinking water and the blood through HPLC system. The level of coumestrol dissolved in the drinking water was almost 10 times higher in the H group than L group. Blood analysis also showed that the coumestrol level was markedly higher in the H group than L group while undetectable in the control mice. These results indicated that the mice in the H and L groups had been successfully exposed to coumestrol as initially designed in this study. At the same time, we have recognized that the exposure level may be more accurately assessed by determination of urinary coumestrol and its metabolite [28, 29]. However, it is quite difficult to collect 24-h urine specimens from the mice for HPLC analysis. We further found that coumestrol exposure had some suppressive effects on thyroid autoimmune responses in the EAT mouse model. Although the intrathyroidal inflammatory score was not significantly altered, serum anti-Tg IgG2a, IgG3 and IgG1 titers were markedly reduced in the animals exposed to low dose of coumestrol (L group) as compared with those of the controls. Since the murine thyroid glands were very small, the extent of the whole mononuclear cell infiltration had been assessed by a rough score system as many studies usually did [15, 16], which may not reflect the changes of one or two T cell subsets among them. The expression pattern of CD4^+^ T subsets and B cells in the spleen were further investigated. CD4^+^ T cells consist of Th and Treg subsets. The former mainly includes Th1, Th2, and Th17. The Th1-mediated autoimmune response is the major pathogenesis of AIT [11]. Th17 has also recently been implicated in thyroid autoimmunity and may contribute to the development of AIT, while Tregs play a protective role from this autoimmune disease [11, 15]. Our study found that the mRNA expression of IFN-γ (Th1 typical cytokine) was significantly decreased, and there was significantly lower proportion of Th1 subset and Th1/Th2 ratio in the splenocytes of L group as compared with those of the control mice. In addition to the direct induction of thyrocyte apoptosis [11], IFN-γ can promote the IgG subclass switching to IgG2a and IgG3, which have more opsonizing and complement-fixing abilities than IgG1 in mice [17, 18]. The ratio of serum anti-Tg IgG2a to IgG1 was markedly lower in the L group than the control. The class-switching of IgG2b is driven by TGFb and IL-17 [30, 31]. In this study, serum anti-Tg IgG2b did not show any significant difference between coumestrol-exposed mice and the control animals. These findings suggested that coumestrol intake might reduce thyroid autoantibody production through suppression of Th1 response in the development of AIT.

Several *in-vitro* studies with cell lines under estrogen-free culture medium have reported that coumestrol treatment reduced the proliferation of T cells and production of TNF-α, IL-1β, IL-6 and IL-2 following polyclonal activation, respectively [32-34]. Moreover, one study showed that coumestrol had a dose-dependent effect on the secretion of IL-2 by CD4^+^ Jurkat T cell line, which was not a purely linear pattern [33]. However, in another study, the secretions of those cytokines were not affected by 5-500 nM coumestrol pre-treatment [35].The effects of coumestrol in the presence of E2 have been studied *in vitro* and found to be concentration-dependent as a double-phase curve [36]. Some other phytoestrogens have also shown U-shaped dose-response curves [37]. They may become ER antagonists at low doses in the presence of endogenous estrogens while act as ER agonists at high doses or in the absence of endogenous estrogens [37]. A few studies have demonstrated that estrogens can enhance IFN-γ production and promote Th1-polarized response [1, 3-5]. ER bound with E2 can directly stimulate IFN-γ mRNA expression through interaction with the estrogen response elements (EREs) in the promoter region of its gene [5]. Our previous study has indicated that ERα is responsible for this action of E2 [1]. The binding affinity and potency of coumestrol to ERα is 10-fold and (10 ~ 100)-fold less than that of E2, respectively [14]. Coumestrol is a full agonist on ERα, and its efficacy on the activation of ERα is similar to that of E2 [14]. In an acute *in-vivo* mammalian assay of uterine vascular permeability, Milligan et al. found that coumestrol required 100-1,000-fold higher doses than E2 [38]. It suggests coumestrol at >1000-fold higher concentration above serum physical E2 level can not only block the binding of endogenous E2 molecules with ER, but also exhibit estrogenic activity as same as E2 does. In this study, serum concentrations of coumestrol in the L and H groups were about 400 and 1200 times higher than that of endogenous E2 on the average, respectively. In the L group, not only the expression of IFN-γ mRNA but also the percentage of Th1 cells was significantly decreased in the splenocytes although T-bet mRNA expression was not changed. The uterine weight was also significantly reduced in the L group as compared with vehicle-treated intact female control. These findings suggested that low dose of coumestrol exposure in the presence of endogenous E2 may suppress IFN-γ production through directly anti-estrogenic activity due to competitive binding with ERα and lower transactivation potency than endogenous E2. However, higher concentration of coumestrol in the H group did not show anti-estrogenic effects possibly due to its similar ERα-agonistic efficacy to that of endogenous E2 although it blocked the binding of E2 with ER. The anti-estrogenic activities have been reported in the studies of the brain and pituitary tissues in OVX mice treated with E2 and autoimmune responses in intact female NZB/NZW F1 mouse model of lupus after they were exposed to non-soy mouse chow containing coumestrol [8, 10] In the latter study, the partially suppressive effects of coumestrol exposure on autoantibody production and target organ damage in NZB/NZW F1 mice were found due to its actions on immune response rather than target organ susceptibility [10]. The actions of coumestrol have been suggested directly through the “classic” genomic ER pathway [9, 14], alternate nongenomic pathway [39] or/and indirectly through inhibition of the conversion of estrone to 17ß-E2 [40]. In this study, coumestrol-treated groups did not show any significant changes in serum E2 levels as compared with the vehicle control. Uterine weight was significantly decreased in the L group. These findings indicated that coumestrol exerted anti-estrogenic actions without direct inhibition of E2 production.

Phytoestrogen exposure due to intake of plant-derived foods from the Chinese market has been investigated including daidzein, genistein, secoisolariciresinol, glycitein and coumestrol [41]. Based on the overall population sampled (*n* = 1000), the average total phytoestrogen intake was estimated at 232 μg/kg/day (equivalent to 42 μg/mouse/day) in which genistein contributed to about 66% while coumestrol was present only in trace amount [41]. In the Study of Women's Health across the Nation (SWAN), Greendale GA et al analyzed dietary phytoestrogens in a cohort of African American, white, Chinese, and Japanese women living in the US [42]. In their study, coumestrol intake was found 10 times greater in Asian (2.5~170.6μg/day) *versus* non-Asian participants (0.4~62.7μg/day), and their highest dietary intake amount of coumestrol was only equivalent to 0.5μg/day in a mouse [42]. Hence, the dose administered to L group (about 4 μg/mouse/day) in our study may be closer to the actual exposure levels in the human being after intake of some plant foods or Chinese medicinal herbs. Administration of coumestrol into ER gene knock-out mice may help to figure out whether such effects are mediated by ER subtypes, especially ERα. However, the established ER subtype gene knockout mice were created from two inbred mouse strains, C57BL/6J (H-2b) and J129 (H-2b), and not from EAT-susceptible mouse strains with H-2k (e.g. CBA/J) [43].

Taken together, our findings indicate that coumestrol treatment can reduce the production of thyroid autoantibodies in the development of EAT through suppression of the Th1 response, which may ameliorate the disease to some extent. The beneficial effects of coumestrol exposure found in our study suggest that intake of certain foods or Chinese herbal medicines rich in coumestrol may contribute to the remission of AIT. The influence of coumestrol exposure on high ER-expressing organs (e.g., breast, uterus and prostate) should be taken into account. Coumestrol exposure at the doses administered in this study did not stimulate uterine growth but rather exerted anti-estrogenic activity, which suggests potentially reduced risk of cancer in the breast and other reproductive organs [44]. One limitation of our study was that the exposure time of coumestrol was relatively short to observe its influence on the progression of AIT. Future studies are also needed to figure out the optimal exposure doses of coumestrol to benefit AIT patients at different ages so that new therapeutic and prophylactic strategies could be developed.

## MATERIALS AND METHODS

### Animals

Five-week-old female CBA/J mice (18-20g) were purchased from Beijing HFK Biotechnology Co. Ltd. (Beijing, China). They were housed under specific-pathogen-free (SPF) standard conditions at 24 ± 2°C with automatic 12-h light and 12-h dark cycles in the animal facility of China Medical University (National Animal Use License number: SCXK-LN2003-0009). This study was approved by the Institutional Animal Care and Use Committee of China Medical University. All experiments and procedures were carried out in accordance with the Guide for the Care and Use of Laboratory Animals mandated by the National Institutes of Health. Food and water were provided *ad libitum*. All mice were fed a phytoestrogen-minimal diet produced by Beijing HFK Co. based on the formula of Rodent Diet 2016 provided by Harlan Laboratories (Chicago, IL, U.S.).

### Coumestrol treatment, analysis, and immunization protocols

One week after their arrival, the mice were randomly divided into three groups, and given the following exposure treatments until they were sacrificed. (1) High coumestrol group (H group): the mice were fed 8 mg/l coumestrol (Sigma, St. Louis, MO, U.S.) in 1% Tween80 dissolved in drinking water. (2) Low coumestrol group (L group): the animals were fed 0.8 mg/l coumestrol in 1% Tween80 in drinking water. (3) Control group: the mice were fed only 1% Tween80 in drinking water. The dosing regimens in this study were selected based on the potential anti-estrogenic effects of coumestrol on the reproductive organs in intact adult animals and estrogenic actions in neonatal and ovariectomized mice found by previous reports [19, 45]. 1% Tween80 was used as dissolvent for coumestrol in drinking water [45].

Successful exposure of the mice to coumestrol was further confirmed by determination of its concentrations in the serum and drinking water using high performance liquid chromatography (HPLC). At first, standard solutions (30-450 ng/ml) were prepared and used to confirm the linear relationship between chromatographic peak area and concentration of coumestrol. Twenty microliters of the sample were injected into a column with methyl alcohol-water (53:47, v/v) as eluent at a flow rate of 1.0 ml/min. Analytes were monitored using a UV-VIS detector at 254 nm. Secondly, puerarin (3 μg/ml in methanol) was used as an internal standard. Finally, the serum samples and coumestrol standard solutions were all extracted with acetonitrile, and re-dissolved in methanol/water (53:47, vol/vol) followed by detection through the HPLC system. The chromatographic peak area was recorded (three injections for each sample). The concentration of coumestrol was calculated using the standard curve according to literatures [6, 29, 46]. In addition, the concentrations of coumestrol in drinking water administered to the mice were confirmed through HPLC system. A 20-μl aliquot of each drinking water sample (*n* = 3) was injected into a column with methyl alcohol-water (53:47, v/v) as eluent at a flow rate of 1.0 ml/min. Analytes were monitored by UV-VIS detector at 254 nm.

Mouse Tg (mTg) was prepared as described in our previous study [15]. In brief, frozen thyroids from Kunming mice bred in the animal facility of China Medical University were homogenized in phosphate-buffered saline (PBS, pH 7.2) at 4°C. After precipitated with 42%, 37%, and 42% saturated ammonium sulfate, mTg was further purified on a Sephadex G-200 column. To induce EAT, the three groups of CBA/J mice were first immunized with mTg (200 μg/mouse) in complete Freund's adjuvant (Sigma, St. Louis, MO, USA) at 9 weeks of age, and then challenged with mTg (200 μg/mouse) in incomplete Freund's adjuvant at 11 weeks of age. Four weeks later, they were sacrificed for assessment of EAT as described previously [16, 47].

### Measurement of anti-Tg autoantibody (TgAb)production

Mice (*n* = 10-14 per group) were anesthetized with isoflurane and bled from the orbital plexus just before sacrificed. Serum samples were stored at −80°C. Serum titers of four IgG subclasses against mTg were detected by ELISA as described in our previous studies [1, 15, 47]. Serially diluted (five-fold) serum samples were added into the 96-well ELISA plates (Nunc) which had been coated with mTg (1 μg/well) and blocked with PBS-1% BSA (Sigma), and then incubated at room temperature for 2h. Anti-Tg IgG subclasses were detected using horseradish peroxidase-conjugated goat anti-mouse IgG1 (1:30,000), IgG2a (1:30,000), IgG2b (1:15,000), or IgG3 (1:250) (all from Bethyl, TX, U.S.). Plates were developed with tetramethylbenzidine (TMB, Amresco, OH, U.S.) and absorbance at 450 nm was measured using a 96-well plate reader (Bio-Rad, CA, U.S.). These determinations were performed in duplicate and their mean values were used for statistical analysis.

### Histological evaluation of EAT

Thyroid glands were removed immediately after Tg-immunized mice (*n* = 14-16 per group) were sacrificed. They were fixed in 10% neutral phosphate-buffered formalin (Sigma), embedded in paraffin, sectioned, and stained with hematoxylin and eosin (HE) for histological examination. The extent of mononuclear cell infiltration was observed under a light microscope (BX51/BX52, Olympus, Tokyo, Japan) and then scored as previously reported by Verginis et al. in a blinded fashion [16]. Score 0 = no infiltration; 1 = interstitial accumulation of cells between two or three follicles; 2 = one or two foci of cells at least the size of one follicle; 3 = extensive infiltration 10-40% of total area; 4 = extensive infiltration 40-80% of total area; and 5 = extensive infiltration >80% of total area.

### Tissue collection and single-cell preparation

In addition to blood and thyroid specimens, the spleen, ovary, and uterus were also immediately removed from the above mice at the time of sacrifice. Some spleens were snap-frozen in liquid nitrogen and kept at -70ºC. Some (*n* = 4/group) were removed, weighed, and individually dissociated into single-cell suspensions through 100-μm nylon mesh. Splenic mononuclear cells (i.e., splenocytes) were further isolated by density gradient centrifugation with Lympholyte-M (Accurate Chemical & Scientific Co., Westbury, NY, U.S.). Then the splenocytes were washed, re-suspended, counted *via* hematocytometer (Clay-Adams, New York, NY, U.S.), and aliquoted for immunofluorescent staining as described in our previous study [1]. Besides, splenocytes from 4 to 6 representative mice per group were sterilely isolated and re-suspended in phenol-red free complete RPMI-1640 medium (Invitrogen Co., Carlsbad, CA, U.S.) with 10% charcoal-stripped fetal bovine serum (FBS; Sigma) and then aliquoted for cell culture followed by immunofluorescent staining. Ovaries and uteri were weighed after removal of associated fat and expression of any internal fluid. Body weight was monitored once every 2 weeks. The organ weights are presented as relative organ weights after normalized to the body weights using the following equation: organ weight (mg)/body weight (g).

### Immunofluorescent staining and flow cytometry

Aliquots of splenocytes (1×10^6^) (*n* = 4-6 per group) were first preblocked with purified anti-Fcγ III/II receptor (clone 2.4G2, BD PharMingen) to eliminate nonspecific binding to Fc receptors. Dual immunofluorescent surface staining was performed on some splenocytes with anti-IgM-FITC (clone R6-60.2, BD PharMingen) and anti-CD45R/B220-PE (clone RA3-6B2, BD PharMingen) to analyze B cells as described in our previous study [1]. Sterilely isolated splenocytes were further stimulated with phorbol myristate acetate (PMA) plus ionomycin (Sigma-Aldrich) in the presence of brefelid-A (Sigma-Aldrich) at 37°C for 5 h. Those cells were then stained with anti-CD4-FITC (clone RM4-5, eBioscience, San Diego, CA, U.S.) and anti-IFN-γ-PE (clone XMG1.2, BD PharMingen) for T helper (Th)1 cells and with anti-CD4-FITC and anti-IL-4-PE (clone 11B11, BD PharMingen) for Th2 cells according to the manufacturer's instructions for dual immunofluorescent surface/intracellular staining. Isotype controls Abs (BD PharMingen) were used as negative controls. Finally, the cells were analyzed on a FACScan flow cytometer using CellQUEST software (Becton-Dickinson, CA, U.S.). Debris and dead cells were excluded by gating based on forward scatter and side scatter parameters.

### Quantitative RT-PCR analysis

Fresh spleen specimens were immediately removed from the mice mentioned above (*n* = 5-8 per group) at the time of sacrifice, snap-frozen in liquid nitrogen, and then were stored at -70°C until processed. Total RNA was extracted with TRIzol reagent (Invitrogen) and reverse-transcribed into cDNA using a PrimeScript^TM^ RT Reagent Kit (Takara Biotechnology Co., Dalian, China) according to the manufacturer's instructions. Real-time quantitative PCR analysis was performed using a Rotor-Gene 3000 Real-time PCR System (Corbett Research Inc., Mortlake NSW, Australia) with SYBR^®^ Premix Ex Taq^TM^ II (Takara) and primers specific for murine T-box transcription factor 21 (T-bet), GATA-binding protein 3 (GATA3), retinoic acid-related orphan nuclear receptor γt (RORγt), forkhead box protein 3 (Foxp3), interferon (IFN)-γ, interleukin (IL)-4, IL-17A, IL-21, and transforming growth factor (TGF)-β. These are typical cytokines and specific transcription factors to T helper (Th) cells, including Th1 (T-bet and IFN-γ), Th2 (GATA3 and IL-4) and Th17 (RORγt, IL-17A and IL-21), and regulatory T cells (Tregs; Foxp3 and TGF-β), respectively. Primers were designed and synthesized by Takara (Table 1). The transcript levels were normalized to that of murine GAPDH, and expressed as mean ± standard deviation (SD) of △Ct values.

**Table 1:** The primer sequences for quantitative polymerase chain reaction

Gene	Sequence sense and anti-sense
T-box transcription factor 21 (T-bet)	5′- GTTCAACCAGCACCAGACAGAG-3′ 5′- TGGTCCACCAAGACCACATC-3′
Interferon (IFN)-γ	5′-CGG CAC AGT CAT TGA AAG CCTA-3′ 5′-GTT GCT GAT GGC CTG ATT GTC-3′
GATA-binding protein 3 (GATA3)	5′- GGA TGT AAG TCG AGG CCC AAG-3′ 5′- ATT GCA AAG GTA GTG CCCGGTA-3′
Interleukin (IL)-4	5′- ACG GAG ATG GAT GTG CCA AAC-3′ 5′- AGC ACC TTG GAA GCC CT CAGA-3′
Retinoic acid-related orphan nuclear receptor γt (RORγt)	5′- TCTGCAAGACTCATCGACAAGG-3′ 5′-CACATGTTGGCTGCACAGG-3
IL-17	5′- GGAAAGCTGGACCACACCA-3′ 5′-CACACCCACACGCATCTTCTC-3′
Forkhead box protein 3 (Foxp3)	5′- CAC CCA GGA AAG ACA GCA ACC-3′ 5′- CAA GAG CTC TTG TCC TTG A-3′
Transforming growth factor (TGF)-β	5′- GTG TGG AGC AAC ATG TGG AACTCTA-3 5′- CGC TGA ATC GAA AGC CCT GTA-3′
GAPDH	5′- ACT CCA CTC ACG GCA AAT TC -3 5′- TCT CCA TGG TGG TGA AGA CA -3′

### Measurement of serum estradiol (E2) level

Serum E2 levels from 10 to 12 mice per group were determined using an EIA Kit (Cayman Chemical, Ann Arbor, MI, U.S.) according to the manufacturer's instructions.

### Statistical analysis

Data were analyzed with SPSS software (version 16.0, SPSS Inc., Chicago, IL, U.S.). Data are shown as mean ±SD. Comparisons between groups were performed using one-way ANOVA followed by Bonferroni's *post hoc* test, Kruskal-Wallis test, and Chi-square test. Differences were considered statistically significant if *P* < 0.05.

## References

[1] Li J, McMurray RW (2006). Effects of estrogen receptor subtype-selective agonists on immune functions in ovariectomized mice. Int Immunopharmacol.

[2] Li J, McMurray RW (2007). Effects of estrogen receptor subtype-selective agonists on autoimmune disease in lupus-prone NZB/NZW F1 mouse model. Clin Immunol.

[3] Li J, McMurray RW (2010). Effects of cyclic *versus* sustained estrogen administration on peripheral immune functions in ovariectomized mice. Am J Reprod Immunol.

[4] Li H, Li J (2015). Thyroid disorders in women. Minerva Medica.

[5] Khan D, Ansar Ahmed S (2015). The Immune System Is a Natural Target for Estrogen Action: Opposing Effects of Estrogen in Two Prototypical Autoimmune Diseases. Front Immunol.

[6] Hutabarat LS, Greenfield H, Mulholland M (2001). Isoflavones and coumestrol in soybeans and soybean products from Australia and Indonesia. J Food Compos Anal.

[7] Wu CT, Zeng JN, Lai JN, Tsan SH, Wang JD (2012). Prescription profile of Chinese herbal products containing coumestrol, genestein, and/or daidzein among female users: an analysis of national health insurance data in Taiwan between 1997 and 2007. Chin Med.

[8] Jacob DA, Temple JL, Patisaul HB, Young LJ, Rissman EF (2001). Coumestrol antagonizes neuroendocrine actions of estrogen *via* the estrogen receptor alpha. Exp Biol Med (Maywood).

[9] Chandsawangbhuwana C, Baker ME (2014). 3D models of human ERalpha and ERbeta complexed with coumestrol. Steroids.

[10] Schoenroth LJ, Hart DA, Pollard KM, Fritzler MJ (2004). The effect of the phytoestrogen coumestrol on the NZB/W F1 murine model of systemic lupus. J Autoimmun.

[11] Zaletel K, Gaberscek S (2011). Hashimoto's Thyroiditis: From Genes to the Disease. Curr Genomics.

[12] Noel R, Rose (2011). The Genetics of Autoimmune Thyroiditis: the first decade. J Autoimmun.

[13] Guo XQ, Tian EJ, Zhang JL, Zhao SJ, Wang JL (2004). The effects of estrogen on experimental autoimmune thyroiditis in rats. Chinese Journal of Endocrinology and Metabolism.

[14] Mueller SO, Simon S, Chae K, Metzler M, Korach KS (2004). Phytoestrogens and their human metabolites show distinct agonistic and antagonistic properties on estrogen receptor alpha (ERalpha) and ERbeta in human cells. Toxicol Sci.

[15] Xue H, Wang W, Li Y, Shan Z, Teng X, Gao Y, Fan C, Teng W (2010). Selenium upregulates CD4(+)CD25(+) regulatory T cells in iodine-induced autoimmune thyroiditis model of NOD. H-2(h4) mice. Endocr J.

[16] Verginis P, Li HS, Carayanniotis G (2005). Tolerogenic semimature dendritic cells suppress experimental autoimmune thyroiditis by activation of thyroglobulin-specific CD4+CD25+ T cells. J Immunol.

[17] Snapper CM, Paul WE (1987). Interferon-gamma and B cell stimulatory factor-1 reciprocally regulate Ig isotype production. Science.

[18] Snapper CM, McIntyre TM, Mandler R, Pecanha LM, Finkelman FD A, Lees J, Mond J (1992). Induction of IgG3 secretion by interferon gamma: a model for T cell-independent class switching in response to T cell-independent type 2 antigens. J. Exp. Med.

[19] Fredricks GR, Kincaid RL, Bondioli KR, Wright RW (1981). Ovulation rates and embryo degeneracy in female mice fed the phytoestrogen coumestrol. Proc Soc Exp Biol Med.

[20] Okayasu I, Kong YM, Rose NR (1981). Effect of castration and sex hormones on experimental autoimmune thyroiditis. Clin. Immunot. Immunopathol.

[21] Chailurkit LO, Aekplakorn W, Ongphiphadhanakul B (2014). The relationship between circulating estradiol and thyroid autoimmunity in males. Eur J Endocrinol.

[22] Yakimchuk K, Jondal M, Okret S (2013). Estrogen receptor alpha and beta in the normal immune system and in lymphoid malignancies. Mol Cell Endocrinol.

[23] Li J, McMurray RW (2009). Effects of chronic exposure to DDT and TCDD on disease activity in murine systemic lupus erythematosus. Lupus.

[24] Zandman-Goddard G, Solomon M, Rosman Z, Peeva E, Shoenfeld Y (2012). Environment and lupus-related diseases. Lupus.

[25] Li Y, Luh CJ, Burns KA, Arao Y, Jiang Z, Teng CT, Tice RR, Korach KS (2013). Endocrine-Disrupting Chemicals (EDCs): *In Vitro* Mechanism of Estrogenic Activation and Differential Effects on ER Target Genes. Environ Health Perspect.

[26] Hong YH, Huang CJ, Wang SC, Lin BF (2009). The ethyl acetate extract of alfalfa sprout ameliorates disease severity of autoimmune-prone MRL-lpr/lpr mice. Lupus.

[27] Sawai C, Anderson K, Walser-Kuntz D (2003). Effect of bisphenol A on murine immune function: modulation of interferon-gamma, IgG2a, and disease symptoms in NZB X NZW F1 mice. Environ Health Perspect.

[28] Valentin-Blasini L, Blount BC, Caudill SP, Needham LL (2003). Urinary and serum concentrations of seven phytoestrogens in a human reference population subset. J Expo Anal Environ Epidemiol.

[29] Ferreira-Dias G, Botelho M, Zagrajczuk A, Rebordao MR, Galvao AM, Bravo PP, Piotrowska-Tomala K, Szostek AZ, Wiczkowski W, Piskula M, Fradinho MJ, Skarzynski DJ (2013). Coumestrol and its metabolite in mares' plasma after ingestion of phytoestrogen-rich plants: potent endocrine disruptors inducing infertility. Theriogenology.

[30] McIntyre TM, Klinman DR, Rothman P, Lugo M, Dasch JR, Mond JJ, Snapper CM (1993). Transforming growth factor beta 1 selectivity stimulates immunoglobulin G2b secretion by lipopolysaccharide-activated murine B cells. J Exp Med.

[31] Shibui A, Shimura E, Nambu A, Yamaguchi S, Leonard WJ, Okumura K, Sugano S, Sudo K, Nakae S (2012). Th17 cell-derived IL-17 is dispensable for B cell antibody production. Cytokine.

[32] Karieb S, Fox SW (2013). Suppression of T cell-induced osteoclast formation. Biochem Biophys Res Commun.

[33] Ndebele K, Tchounwou PB, McMurray RW (2004). Coumestrol, bisphenol-A, DDT, and TCDD modulation of interleukin-2 expression in activated CD+4 Jurkat T cells. Int J Environ Res Public Health.

[34] Jantaratnotai N, Utaisincharoen P, Sanvarinda P, Thampithak A, Sanvarinda Y (2013). Phytoestrogens mediated anti-inflammatory effect through suppression of IRF-1 and pSTAT1 expressions in lipopolysaccharide-activated microglia. Int Immunopharmacol.

[35] Hong YH, Chao WW, Chen ML (2009). Ethyl acetate extracts of alfalfa (Medicago sativa L.) sprouts inhibit lipopolysaccharide-induced inflammation *in vitro* and *in vivo*. J Biomed Sci.

[36] Wang C, Kurzer MS (1998). Effects of phytoestrogens on DNA synthesis in MCF-7 cells in the presence of estradiol or growth factors. Nutr Cancer.

[37] Almstrup K, Fernández MF, Petersen JH, Olea N, Skakkebaek NE, Leffers H (2002). Dual effects of phytoestrogens result in u-shaped dose-response curves. Environ Health Perspect.

[38] Milligan SR, Balasubramanian AV, Kalita JC (1998). Relative potency of xenobiotic estrogens in an acute *in vivo* mammalian assay. Environ Health Perspect.

[39] Jeng YJ, Watson CS (2009). Proliferative and anti-proliferative effects of dietary levels of phytoestrogens in rat pituitary GH3/B6/F10 cells-the involvement of rapidly activated kinases and caspases. BMC Cancer.

[40] Makela S, Poutanen M, Lehtimaki J, Kostian ML, Santti R, Vihko R (1995). Estrogen-specific 17 beta-hydroxysteroid oxidoreductase type 1 (E.C. 1.1.1.62) as a possible target for the action of phytoestrogens. Proc Soc Exp Biol Med.

[41] Hu XJ, Song WR, Gao LY, Nie SP, Eisenbrand G, Xie MY (2014). Assessment of dietary phytoestrogen intake *via* plant-derived foods in China. Food Addit Contam Part A Chem Anal Control Expo Risk Assess.

[42] Maron R, Cohen IR (1980). H-2K mutation controls immune response phenotype of autoimmune thyroiditis. Critical expression of mutant gene product in both thymus and thyroid glands. J Exp Med.

[43] Greendale GA, Huang MH, Leung K, Crawford SL, Gold EB, Wight R, Waetjen E, Karlamangla AS (2012). Dietary phytoestrogen intakes and cognitive function during the menopausal transition: results from the Study of Women's Health Across the Nation Phytoestrogen Study. Menopause.

[44] Shi L, Xia TS, Wei XL, Zhou WB, Xue JQ, Cheng L (2015). Estrogen receptor (ER) was regulated by RNPC1 stabilizing mRNA in ER positive breast cancer. Oncotarget.

[45] Markaverich BM, Webb B, Densmore CL, Gregory RR (1995). Effects of coumestrol on estrogen receptor function and uterine growth in ovariectomized rats. Environ Health Perspect.

[46] Argenta DF, Franco C, Koester LS, Bassani VL, Teixeira HF (2011). LC analysis of coumestrol incorporated into topical lipid nanoemulsions. Pharmazie.

[47] Guo D, Li L, Liu M, Li J, Shan ZY, Teng WP (2011). Establishment of experimental autoimmune thyroiditis in CBA/J mice by different species and dosages of thyroglobulin. Journal of China Medical University.

